# Cochrane Systematic Reviews of Chinese Herbal Medicines: An Overview

**DOI:** 10.1371/journal.pone.0028696

**Published:** 2011-12-09

**Authors:** Jing Hu, Junhua Zhang, Wei Zhao, Yongling Zhang, Li Zhang, Hongcai Shang

**Affiliations:** 1 Research Institute of Traditional Chinese Medicine, Tianjin University of Traditional Chinese Medicine, Tianjin, China; 2 Department of Epidemiology and Biostatistics, School of Public Health, Peking University, Beijing, China; Finnish Institute of Occupational Health, Finland

## Abstract

**Objectives:**

Our study had two objectives: a) to systematically identify all existing systematic reviews of Chinese herbal medicines (CHM) published in Cochrane Library; b) to assess the methodological quality of included reviews.

**Methodology/Principal Findings:**

We performed a systematic search of *the Cochrane Database of Systematic Reviews* (CDSR, Issue 5, 2010) to identify all reviews of CHM. A total of fifty-eight reviews were eligible for our study. Twenty-one of the included reviews had at least one Traditional Chinese Medicine (TCM) practitioner as its co-author. 7 reviews didn't include any primary study, the remaining reviews (n = 51) included a median of 9 studies and 936 participants. 50% of reviews were last assessed as up-to-date prior to 2008. The questions addressed by 39 reviews were broad in scope, in which 9 reviews combined studies with different herbal medicines. For OQAQ, the mean of overall quality score (item 10) was 5.05 (95% CI; 4.58-5.52). All reviews assessed the methodological quality of primary studies, 16% of included primary studies used adequate sequence generation and 7% used adequate allocation concealment. Of the 51 nonempty reviews, 23 reviews were reported as being inconclusive, while 27 concluded that there might be benefit of CHM, which was limited by the poor quality or inadequate quantity of included studies. 58 reviews reported searching a median of seven electronic databases, while 10 reviews did not search any Chinese database.

**Conclusions:**

Now CDSR has included large numbers of CHM reviews, our study identified some areas which could be improved, such as almost half of included reviews did not have the participation of TCM practitioners and were not up-to-date according to Cochrane criteria, some reviews pooled the results of different herbal medicines and ignored the searching of Chinese databases.

## Introduction

Traditional Chinese Medicine (TCM) is an essential part of the healthcare system in several Asian countries, and is considered a complementary or alternative medical system in most Western countries [1]. Chinese herbal medicines (CHM) are an essential part of TCM [2]. The 2002 National Health Interview Survey showed that 18.6% of adults used CHM in the United States, while it was 12.1% in 1997 [3]. With the increased use of CHM, questions arise from clinicians, patients, and policymakers as to the effectiveness of these interventions [4]. In an era of evidence-based healthcare, systematic reviews of randomized controlled trials (RCTs) are becoming increasingly important as a source of evidence for decision-making. As the number of systematic reviews of CHM increase, the quality of which has been highlighted and called into question. Some studies have assessed the quality of CHM reviews published in Chinese journals, in general, they have been criticized for lacking a comprehensive search for clinical trials, ignoring the characteristics of TCM, using inappropriate criteria to assess the methodological quality of included studies, and addressing too broadly defined questions [5]–[7]. All [8] these aspects could have contributed to a poor quality review.

The Cochrane Collaboration is an international organization that aims to prepare and maintain rigorous systematic reviews in order to help people make well-informed decisions about health care [8]. Compared with reviews published in paper-based journals, Cochrane reviews are noted to have greater methodological quality [9]. Ever since 1999 when the first Cochrane review of CHM was published, a sharp increase has been observed in the number of similar reviews. However, no previous studies have systematically assessed the methodological quality of Cochrane reviews of CHM. Therefore, we did this overview of systematic reviews. Our study had two objectives: a) to systematically identify all existing Cochrane reviews of CHM; b) to assess the methodological quality of included reviews.

## Methods

### Ethics

Data for this study was acquired through previously published work, no patient or hospital data was accessed. Therefore, written consent and institutional ethical review was not required for this research. The PRISMA checklist and flow diagram are available as supporting information; see PRISMA Checklist S1 and PRISMA Flow Diagram S1.

### Literature search

In order to identify reviews focusing on CHM, we searched the titles and abstracts of all reviews contained within *the Cochrane Database of Systematic Reviews* (CDSR) (Issue 5, 2010) using the following terms: *Chinese* or herb* or *traditional* or *plant* or medic*.

### Inclusion and exclusion criteria

We included all Cochrane reviews of CHM. Protocols and reviews which have been withdrawn from publication were excluded. We defined CHM as preparations derived from plants or parts of plants (e.g. leaves, stems, buds, flowers, roots or tubers) that grow in China and have been widely used for medical purpose. CHM include single herbs (or extracts from single herbs) and compound formulas of several herbs in all forms of preparation formulation (e.g. oral liquid, tablet, capsule, pill, powder, plaster or injection liquid). It should be noted that our definition of CHM does not include plant-derived chemicals or synthetic chemicals which contain constituents of plants. For example, although Huperzia serrata has its origin in China, according to our definition, Huperzine A does not belong to CHM because it is a kind of alkaloid extracted from Huperzia serrata. In addition, we only included reviews discussing herbs which originated from China, reviews on herbs such as Passiflora and Echinacea, both of American origin, were invariably excluded.

### Oxman-Guyatt Overview Quality Assessment Questionnaire (OQAQ) [10]

The OQAQ instrument was selected as the quality appraisal tool, which was designed to evaluate whether the authors of a systematic review conducted a comprehensive search, minimized bias in the selection of primary studies, evaluated the primary literature, and pooled the results appropriately. It consists of 10 questions, the first 9 questions are designed to assess different aspects of methodological quality and have set answers of “yes”, “partially/can't tell”, or “no”, question 10 is an assessment of the overall scientific quality of the systematic review on a scale of 1 to 7, it is answered based on how well the review scored on the first 9 questions.

### Data extraction

We established a database (using Microsoft Excel 2007) to extract data. The database had two components: 1) general characteristics, including country of first author and number of authors, whether the review had the participation of TCM practitioners, number of trials and participants included, disease, the year of review last assessed as up-to-date, conclusions drawn by the reviewers (by assessing the reviewers' abstract conclusions statements), interventions in experimental groups, number of herbs included, and whether the results of different herbal medicines were pooled; 2) methodological quality of included reviews, including OQAQ scale, the approach to assessment of methodological quality of primary studies, the number of trials with adequate sequence generation and allocation concealment, and type and number of English and Chinese databases searched. Two reviewers (Jing Hu and Wei Zhao) independently extracted the information of each review, disagreements between the two reviewers were resolved by discussion.

The questions addressed by a review may be broad or narrow in scope, each review was assigned into one of the following two categories: 1) narrowly focused reviews, intervention in each review was single herb or herbal preparation, as an example of “Chinese herbal medicine suxiao jiuxin wan for angina pectoris”; 2) broadly focused reviews, including reviews concerned multiple Chinese herbs or a family of herbal medicines sharing similar efficacy, such as “Chinese herbal medicine for premenstrual syndrome” and “Chinese herbal medicine Huangqi type formulations for nephrotic syndrome”. For broadly focused reviews, we listed the number of herbs included and assessed whether the results of different herbal medicines were pooled.

A review was believed to have the participation of TCM practitioners if at least one author works in TCM department, university or hospital, or it stated that it had got suggestion from TCM practitioners.

When we assessed the type and number of English and Chinese databases searched, we only listed the databases which at least 4 reviews searched. In addition, the Cochrane Specialized Register and databases/websites for ongoing trials were also searched in some reviews, we did not list them in our study.

## Results

278 potentially relevant reviews were obtained, after selection (according to inclusion and exclusion criteria), a total of 58 Cochrane reviews [11]–[68] were eligible for our study, a full list of reviews is included in Table S1. Of the 58 reviews, one review [49] included herbs originated in China, India and Japan; interventions in another review [55] concerned both herbal and chemical medicines. In these two cases, we extracted and analyzed the information relating to CHM.

### General characteristics of included reviews

The number of authors in the 58 reviews ranged from 1 to 10, the first authors were most often from China (46 [79%]), followed by UK (n = 8), and Netherlands, Canada, USA and Australia each have one first-authored review. Twenty-one (36%) of the included reviews had at least one TCM practitioner as its co-author. 7 (12%) reviews didn't include any primary study, of the remaining reviews (n = 51), a total of 671 studies and 75,609 participants were included, the median number of studies and participants included were 9 (Quartile: 3, 15) and 936 (Quartile: 492, 1567) respectively. 50% of the reviews were last assessed as up-to-date prior to 2008, of reviews considered out-of-date, one was last updated in 2000. In total, 44 diseases were investigated in the included reviews, 18 (31%) reviews addressed cerebral vascular and cardiovascular diseases (9 reviews focused on stroke), followed by reviews focused on respiratory diseases (n = 6) and gynecological/pregnancy diseases (n = 6).

Of the 51 nonempty reviews, only one review concluded positively, 27 (53%) concluded that there might be benefit of CHM for treating specific health conditions, which was limited by the poor quality or inadequate quantity of studies, 23 (45%) reviews concluded that the currently available data do not allow any conclusion to be drawn, generally because of low methodological quality of studies, small number of studies and participants included or publication bias.

Nineteen reviews focused on 13 single herbs or herbal preparations, while the remainder (39 [67%]) addressed broad questions, in which 34 reviews concerned multiple Chinese herbs or multiple formulations of Chinese herbs, 5 reviews involved a family of herbal medicines sharing similar efficacy, including Huangqi type formulations (including Huangqi injection and Huangqi-Danggui mixture), Chuanxiong preparations (including Nao-an capsule, Xifeng wan and Apoplexy Preventing Dry Ointment Powder), Dan Shen agents (including Compound Danshen Dripping Pill, Compound Danshen injection, Danshen injection, Yiqi huoxue injection, and Quyu huatan xiezhuo fang) and Sanchi (including Xinnaotai, Sanchitongshu capsule, Naoming injection, Xuesaitong soft capsule, Sanqitongshu capsule, Xuesaitong and Xueshuantong injection). Of the 39 reviews, 4 didn't include any primary study, of the remaining reviews (n = 35), the median number of herbal medicines involved was 6 within a range of 1 to 71, results of different herbal medicines were pooled in 9 reviews, in which 7 reviews pooled the results of all Chinese herbal medicines, one review pooled all Danshen agents, and one pooled Sanchi.

### Methodological quality of included reviews

Table 1 presents a summary of OQAQ items of the included reviews, the mean score (item 10) was 5.05, 95% CI (4.58, 5.52). 41 of 58 reviews attempted to minimize bias during the selection of studies by at least two reviewers independently select eligible studies.

**Table 1:** Summary of OQAQ questions in included reviews.

OQAQ question	Yes(%)	Partially/Can't tell(%)	No(%)
1. Were the search methods used to find evidence reported?	58(100)	0(0)	0(0)
2. Was the search strategy for evidence reasonably comprehensive?	51(88)	1(2)	6(10)
3. Were the criteria used for deciding which studies to include reported?	58(100)	0(0)	0(0)
4. Was bias in the selection for studies avoided?	41(71)	13(22)	4(7)
5. Were the criteria used for assessing validity of included studies reported?	57(98)	1(2)	0(0)
6. Was the validity of included studies assessed using appropriate criteria?	56(97)	2(3)	0(0)
7. Were the methods used to combine the findings of studies reported?	49(84)	9(16)	0(0)
8. Were the Findings of studies combined appropriately?	48(83)	9(15)	1(2)
9. Were the conclusions made by authors supported by the reported data?	58(100)	0(0)	0(0)
10. How would you rate the scientific quality of this overview?	5.05(1.78)*, (95% CI: 4.58, 5.52)		

OQAQ, Oxman-Guyatt Overview Quality Assessment Questionnaire.

*Mean (SD).

All reviews reported assessing the methodological quality of included primary studies, 21 (36%) reviews used the Cochrane Collaboration's ‘Risk of Bias’ tool, the Jadad scale was used in 6 reviews, 12 (21%) reviews used unnamed checklist. Among 671 included studies, 108 (16%) used adequate sequence generation, allocation concealment was adequate in 50 (7%) studies.

The median number of databases searched in 58 reviews was 7 within a range of 4 to 15. Regarding to the English language databases, the most searched was MEDLINE (98%), followed by EMBASE (97%) and CENTRAL (97%). CBM was the most searched Chinese database (78%), the second most used was CNKI (45%) and the third was VIP (24%) (Table 2). All reviews searched at least 2 English databases, while 10 reviews did not search any Chinese database. 41 (71%) reviews searched at least 3 English databases, while only 6 (10%) reviews searched at least 3 Chinese databases (Table 3).

**Table 2:** Databases searched in included reviews.

English databases searched	Number of reviews (%) n = 58	Chinese databases searched	Number of reviews (%) n = 58
MEDLINE*	57(98%)	CBM(Chinese Biomedical Database)	45(78%)
CENTRAL(The Cochrane Central Register of Controlled Trials)	56(97%)	CNKI(China National Knowledge Infrastructure)	26(45%)
EMBASE	56(97%)	VIP (a full text database of China)	14(24%)
AMED(Allied and Complementary Medicine Database)	28(48%)	The Chinese Cochrane Centre Controlled Trials Register	11(19%)
Cochrane Complementary Medicine Field Trials Register	20(34%)	TCMLARS(Traditional Chinese Medical Literature Analysis and Retrieval System)	6(10%)
CINAHL(Cumulative Index to Nursing and Allied Health Literature)	15(26%)		
LILACS(Latin American and Caribbean Health Science Literature)	11(19%)		
SIGLE(System for Information on Grey Literature in Europe)	7(12%)		
PsycINFO	4(7%)		

*including two reviews which used PubMed.

**Table 3:** Number of English and Chinese databases searched.

Number of English databases	Number of reviews (%) n = 58	Number of Chinese databases	Number of reviews (%) n = 58
0	0(0%)	0	10(17%)
1	0(0%)	1	15(26%)
2	2(3%)	2	19(33%)
3	15(26%)	3	8(14%)
>3	41(71%)	>3	6(10%)

## Discussion

Evidence-based health care involves the systematic collection, synthesis and application of scientific evidence to guide clinical practice and policy-making. Systematic reviews are a key component of evidence-based health care. Currently, CDSR has included 58 systematic reviews of CHM.

Almost seventy percent of included reviews' topics were too broad, the percentage was much higher than that of similar reviews published in Chinese journals, which was 38 percent (41 among 107 reviews) [69]. It is difficult to develop a comprehensive search strategy for broadly focused reviews, for instance, one hundred and sixty herbal medicines are now available for coronary heart diseases treatment, a systematic review of CHM for coronary heart disease will have to include clinical trials of all these herbal medicines, it is easily to cause the incomplete identification of relevant studies. Choosing broad topics for reviews will require more resources in data collection and analysis, the results may also be too complicated to interpret. So topic selection of CHM reviews should focus on specific clinical problems, the extensive titles are not recommended.

As broad questions of reviews may be addressed by large sets of heterogeneous studies, the data synthesis may be particularly challenging. In our study, 9 reviews (among the 39 broadly focused reviews) pooled the results of different herbs, which did not identify potentially important differences in effects across different interventions. Systematic reviews can, but do not have to use meta-analysis when combining data from primary studies, prior to conducting a meta-analysis, reviewers should examine the consistency of the interventions. It is recommended that the data of each intervention should be analyzed and presented separately if several different interventions for the same condition were tested in one review.

The goal of a systematic review is to identify relevant studies completely and unbiasedly [70]. It has been demonstrated [71], [72] that significant amounts of evidence would potentially be missed if the search is limited to English-only sources. Because a considerable number of clinical trials on CHM were published only in the Chinese language journals, so a comprehensive search of Chinese databases is essential for a systematic review of CHM. However, we were disappointed to find that almost twenty percent of reviews did not search any Chinese database in our study. One study [73] compared four Chinese databases and concluded that CBM is the preferred database for systematic reviewers to retrieve relevant Chinese studies, while CNKI is recommended for non-Chinese-speaking researchers due to its free searched English version website (www.global.cnki.net) and “Cross-Language Search” functions. CBM has no English website and a fee is charged for searching, now many Chinese medical universities have got the permission to search CBM, so maybe the most cost-and time-efficient way to search it is to enhance collaboration with Chinese researchers.

In doing a review of CHM, professional advice from TCM practitioners is of great value. It is generally assumed that the characteristics of TCM would be well taken into account in a review if one or some of its reviewers majored in TCM. In our study, we found that more than sixty percent of reviews did not have one TCM practitioner in the authors list, which might lead to insufficient consideration of the characteristics of TCM (e.g. determination of treatment based on pathogenesis obtained through differentiation of symptoms and signs) and incorrect results. We suggest that future reviews should be authored by a group of individuals with both clinical expertise and methodological expertise.

Of the 671 primary studies included, less than twenty percent of studies used adequate sequence generation, and only seven percent used adequate allocation concealment. Because of the poor methodological quality of primary studies, nearly half of reviews were reported as being inconclusive, while 27 reviews provided preliminary evidence of CHM's benefits to certain conditions, which should be considered tentative and need to be confirmed with rigorous RCTs. The Chinese government has been aware of the importance of conducting scientifically sound RCTs and has made substantial investments into funding clinical researches of CHM, now many well-designed RCTs of CHM with rigorous methodology are in progress or have been completed in China [74], we believe future updates of currently inconclusive Cochrane reviews of CHM may reach more definitive conclusions.

### Limitations of the study

Although we believed a review has a greater chance of considering the characteristics of TCM if at least one author works in TCM department, university or hospital, or it stated that it had got suggestion from TCM practitioners, it is quite possible that some reviewers had consulted TCM experts while designing and doing the review, but did not report it in articles. As cases of this kind could not be ruled out, we therefore might have underestimated the proportion of reviews getting support from TCM practitioners. In addition, we restricted our search to Cochrane reviews because they are generally less prone to bias than systematic reviews published in paper-based journals [9], [75], [76]. However, this might cause the results of this study to be only applicable to review articles in the Cochrane database. Further evaluation is needed in order to know whether the systematic reviews of CHM published in English paper-based journals even in the leading journals have the same problem.

## Supporting Information

Table S1 List of all included Cochrane reviews of CHM.
(DOC)

PRISMA Checklist S1 (DOC)

PRISMA Flow Diagram S1 (DOC)
