# Supplementary material for: Cochrane Systematic Reviews of Chinese Herbal Medicines: An Overview
Source: PLoS One. 2011 Dec 9;6(12):e28696. doi: 10.1371/journal.pone.0028696 (PMC3235143; doi:10.1371/journal.pone.0028696)
Supplement: Table S1 — List of all included Cochrane reviews of CHM. (DOC) [file pone.0028696.s001.doc]

Table S1. Characteristics of included 58 reviews

| Systematic review | Trials  (N) | Participants  (N) | Trials with adequate sequence generation (N) (%) | Trials with adequate allocation concealment (N) (%) | Diseases | Interventions | Included herbal medicines (N) | Whether pooled different herbs |
| --- | --- | --- | --- | --- | --- | --- | --- | --- |
| broadly defined reviews (n=39) | | | | | | | | |
| Jianping Liu [11] | 9 | 936 | 2(22) | 1(11) | chronic hepatitis B | Chinese medicinal herbs | 9 | N |
| Weiya Zhang [13] | 4 | 159 | 1(25) | 0(0) | atopic eczema | Chinese herbal medicine | 1 | N/A |
| Xuejun Cui [14] | 4 | 1100 | 0(0) | 0(0) | neck pain | Chinese herbal medicine | 3 | N |
| Flower A [15] | 2 | 158 | 2 (100) | 2 (100) | endometriosis | Chinese herbal medicine | 1 | N/A |
| Zheng Jing [16] | 2 | 549 | 2 (100) | 1 (50) | premenstrual syndrome | Chinese herbal medicine | 2 | N |
| Xiaoshu Zhu [17] | 39 | 3475 | 2(5) | 2(5) | primary dysmenorrhoea | Chinese herbal medicine | 37 | Y |
| Rathbone J [18] | 7 | 1094 | 0(0) | 0(0) | schizophrenia | Chinese herbal medicine | 6 | Y |
| Wei Yuan [19] | 3 | 128 | 3(100) | 0(0) | nephrotic syndrome | Huangqi type formulations | 3 | N |
| Qiong Wang [21] | 15 | 845 | 0(0) | 0(0) | acute pancreatitis | Chinese herbal medicines | 12 | Y |
| Xiaoxi Zeng [22] | 13 | 1770 | 13(100) | 0(0) | hyperthyroidism | Chinese herbal medicines | 13 | N |
| Tao Gan [23] | 55 | 5261 | 3(5) | 0(0) | gastric cancer | Chinese herbal medicines | 46 | N |
| Grant SJ [24] | 16 | 1391 | 11(69) | 5(31) | impaired glucose tolerance or impaired fasting blood glucose | Chinese herbal medicines | 15 | N |
| Wenjuan Li [25] | 0 | 0 | N/A | N/A | pre-eclampsia | Chinese herbal medicines | N/A | N/A |
| Jianping Liu [26] | 66 | 8302 | 7(11) | 0(0) | type 2 diabetes mellitus | Chinese herbal medicines | 69 | N |
| Dengfeng Wang [27] | 2 | 157 | 2(100) | 0(0) | ectopic pregnancy | Chinese herbal medicines | 2 | Y |
| Xuemei Liu [28] | 13 | 654 | 4(31) | 0(0) | severe acute respiratory syndrome | Chinese herbs combined with Western medicine | 13 | N |
| Taixiang Wu [29] | 4 | 342 | 1(25) | 0(0) | colorectal cancer | Chinese medical herbs | 4 | Y |
| Jiafu Wei [30] | 0 | 0 | N/A | N/A | acute bronchitis | Chinese medicinal herbs | N/A | N/A |
| Jianping Liu [31] | 11 | 932 | 2(18) | 1(9) | asymptomatic carriers of hepatitis B virus infection | Chinese medicinal herbs | 8 | Y |
| Xiaoyang Chen [32] | 2 | 1012 | 2(100) | 1(50) | influenza | Chinese medicinal herbs | 2 | N |
| YongLi Zheng [33] | 0 | 0 | N/A | N/A | measles | Chinese medicinal herbs | N/A | N/A |
| Yunying Shi [34] | 7 | 1253 | 7(100) | 0(0) | sore throat | Chinese medicinal herbs | 7 | N |
| Xiaoge Zhang [35] | 17 | 3212 | 17(100) | 7(41) | common cold | Chinese medicinal herbs | 16 | N |
| Mingming Zhang [36] | 7 | 542 | 3(43) | 0(0) | breast cancer | Chinese medicinal herbs | 7 | N |
| Xunzhe Yang [37] | 3 | 5042 | 1(33) | 1(33) | stroke | Chuanxiong preparations | 3 | N |
| Bo Wu [39] | 6 | 494 | 1(17) | 0(0) | acute ischaemic stroke | Danshen agents | 3 | Y |
| Taixiang Wu [40] | 6 | 2368 | 1(17) | 0(0) | acute myocardial infarction | Danshen preparations | 6 | N |
| Arnold E [49] | 3 | 492 | 1(33) | 0(0) | chronic asthma | Herbal interventions | 5 | N |
| Gagnier JJ [50] | 10 | 1567 | 7(70) | 3(30) | back pain | Herbal medicine | 9 | N |
| Jianping Liu [51] | 9 | 499 | 5(56) | 3(33) | HIV infection and AIDS | Herbal medicines | 8 | N |
| Jianping Liu [52] | 75 | 7957 | 4(5) | 3(4) | irritable bowel syndrome | Herbal medicines | 71 | N |
| Jianping Liu [53] | 40 | 3448 | 0(0) | 0(0) | viral myocarditis | Herbal medicines | 25 | N |
| Jianping Liu [54] | 2 | 150 | 2(100) | 1(50) | uterine fibroids | Herbal preparations | 2 | N |
| Hongmei Wu [55] | 1 | 60 | 0(0) | 0(0) | nephrotic syndrome | Interventions | 1 | N/A |
| Jianping Liu [57] | 10 | 517 | 1(10) | 2(20) | hepatitis C virus infection | Medicinal herbs | 10 | N |
| Xiaoyan Chen [60] | 8 | 660 | 8(100) | 3(38) | acute ischaemic stroke | Sanchi | 5 | Y |
| Adams D [64] | 0 | 0 | N/A | N/A | idiopathic chronic fatigue and chronic fatigue syndrome | Traditional Chinese medicinal herbs | N/A | N/A |
| Qifu Li [65] | 5 | 1125 | 0(0) | 0(0) | epilepsy | Traditional Chinese medicine | 5 | N |
| Qi Zhuo [66] | 3 | 216 | 3(100) | 2(67) | stable angina | Traditional Chinese herbal products | 3 | Y |
| narrowly defined reviews (n=19) | | | | | | | | |
| Weizheng Li [12] | 13 | 962 | 1(7) | 0(0) | acute ischaemic stroke | Acanthopanax | N/A | N/A |
| Xin Duan [20] | 15 | 1776 | 0(0) | 0(0) | angina pectoris | Suxiao Jiuxin Wan | N/A | N/A |
| Yong Yuan [38] | 2 | 161 | 2(100) | 0(0) | acute ischemic stroke | Chuanxiong injection | N/A | N/A |
| Wenzhai Cao [41] | 9 | 723 | 0(0) | 0(0) | acute cerebral infarction | Dengzhanhua injection | N/A | N/A |
| Rui Dong [42] | 0 | 0 | N/A | N/A | lung cancer | Elemene | N/A | N/A |
| Evans JR [43] | 2 | 119 | 2(100) | 2(100) | macular degeneration | Ginkgo biloba | N/A | N/A |
| Xianrong Zeng [44] | 10 | 792 | 1(10) | 1(10) | acute ischaemic stroke | Ginkgo biloba | N/A | N/A |
| Birks J [45] | 35 | 4291 | 35(100) | 17(49) | cognitive impairment and dementia | Ginkgo biloba | N/A | N/A |
| Nicolaï SPA [46] | 14 | 739 | 6(43) | 1(7) | intermittent claudication | Ginkgo biloba | N/A | N/A |
| Hilton MP [47] | 3 | 1143 | 3(100) | 3(100) | tinnitus | Ginkgo biloba | N/A | N/A |
| Ruoling Guo [48] | 14 | 1110 | 6(43) | 3(21) | chronic heart failure | Hawthorn extract | N/A | N/A |
| Weimin Yang [56] | 15 | 1280 | 2(13) | 1(7) | acute ischaemic stroke | Mailuoning injection | N/A | N/A |
| Yan Tan [58] | 1 | 98 | 0(0) | 0(0) | acute ischaemic stroke | Puerarin | N/A | N/A |
| Qin Wang [59] | 20 | 1240 | 0(0) | 0(0) | unstable angina pectoris | Puerarin injection | N/A | N/A |
| Jin Chen [61] | 19 | 1663 | 1(5) | 0(0) | heart failure | Shengmai injection | N/A | N/A |
| Taixiang Wu [62] | 18 | 1413 | 1(6) | 0(0) | unstable angina pectoris | Tongxinluo capsule | N/A | N/A |
| Qi Zhuo [63] | 2 | 232 | 0(0) | 0(0) | acute stroke | Tongxinluo capsule | N/A | N/A |
| Taixiang Wu [66] | 0 | 0 | N/A | N/A | vascular dementia | Yizhi capsule | N/A | N/A |
| Jirong Yue [67] | 0 | 0 | N/A | N/A | vascular dementia | Zhiling decoction | N/A | N/A |
