# Supplementary material for: Cochrane Systematic Reviews of Chinese Herbal Medicines: An Overview
Source: PLoS One. 2011 Dec 9;6(12):e28696. doi: 10.1371/journal.pone.0028696 (PMC3235143; doi:10.1371/journal.pone.0028696)
Supplement: PRISMA Flow Diagram S1 — (DOC) [file pone.0028696.s003.doc]

**Identification**

**Screening**

**Eligibility**

**Included**

278 of records identified through database searching

0 of additional records identified through other sources

0 of records after duplicates removed

278 of records screened

201 of records excluded

77 of full-text articles assessed for eligibility

58 of studies included in qualitative synthesis

19 of full-text articles excluded, with reasons

0 of studies included in quantitative synthesis (meta-analysis)
